# Targeting tumor angiogenesis and metabolism with photodynamic nanomedicine

**DOI:** 10.3389/fcell.2025.1558393

**Published:** 2025-04-01

**Authors:** Yong Lv, Lihui Pu, Bei Ran, Bo Xiang

**Affiliations:** ^1^ Department of Pediatric Surgery, West China Hospital, Sichuan University, Chengdu, China; ^2^ Department of Critical Care, West China Hospital, Sichuan University, Chengdu, China; ^3^ School of Biomedical Engineering, Sichuan University, Chengdu, China

**Keywords:** vascular normalization, metabolic reprogramming, tumor hypoxia, photodynamic therapy, nanomedicine

## Abstract

Photodynamic therapy (PDT) holds considerable promise as a tumor treatment modality, characterized by its targeted action, compatibility with other therapeutic approaches, and non - invasive features. PDT can achieve remarkable spatiotemporal precision in tumor ablation through the generation of reactive oxygen species (ROS). Nevertheless, despite its potential in tumor treatment, PDT encounters multiple challenges in practical applications. PDT is highly oxygen - dependent, and thus the effectiveness of PDT can be markedly influenced by tumor hypoxia. The co-existence of abnormal vasculature and metabolic deregulation gives rise to a hypoxic microenvironment, which not only sustains tumor survival but also undermines the therapeutic efficacy of PDT. Consequently, targeting tumor angiogenesis and metabolism is essential for revitalizing PDT. This review emphasizes the mechanisms and strategies for revitalizing PDT in tumor treatment, predominantly concentrating on interfering with tumor angiogenesis and reprogramming tumor cell metabolism. Lastly, the outlining future perspectives and current limitations of PDT are also summarized. This could provide new insights and methodologies for overcoming the challenges associated with PDT in tumor treatment, ultimately advancing the field of PDT.

## 1 Introduction

**FIGURE 1:**
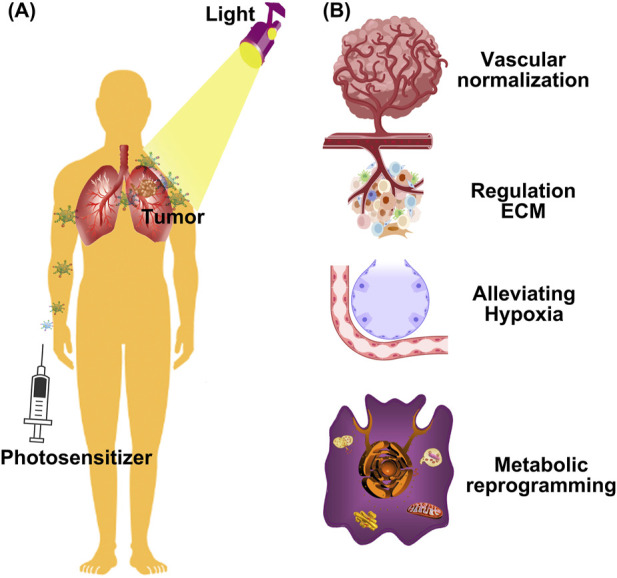
Schematic illustration of different strategies for rejuvenating photodynamic therapy. **(A)** Nanoparticles passively accumulate in tumors due to the enhanced permeability and retention (EPR) effect, where they can be activated with light to produce ROS; **(B)** The latest strategies for rejuvenating PDT, from vascular normalization, regulating extracellular matrix (ECM), and metabolic reprogramming to reverse hypoxia.

Malignant tumors have threatened human health and are one of the major causes of death. Currently, tumor treatment modes in clinical practice are mainly based on surgery, radiation therapy and chemotherapy. However, in many cases, surgical treatment does not achieve complete resection of tumor tissues, and there is a risk of tumor metastasis and recurrence (Raskov et al., 2020). Additionally, radiotherapy and chemotherapy lack tumor specificity, resulting in significant toxic side effects that can damage normal tissues and lead to various adverse reactions in patients (Wang et al., 2024). Therefore, there is an urgent need to develop novel tumor treatment modalities to address the limitations of traditional therapeutic approaches. In recent years, emerging therapeutic modalities such as photodynamic therapy (PDT) have demonstrated significant potential in the treatment of malignant tumors. PDT has garnered attention for its minimal invasiveness, excellent spatiotemporal controllability, and high biocompatibility (Overchuk et al., 2023). PDT utilizes photosensitizers that selectively accumulate in tumor cells. Upon activation by specific light wavelengths, these photosensitizers generate reactive oxygen species (ROS), leading to the destruction of tumor cells. PDT revolutionizes oncological treatment through its cell-selective photochemical reactions, offering new dimensions for therapeutic optimization (Zolyniak-Brzuchacz et al., 2024). We review the latest strategies focused on two primary approaches, regulating tumor angiogenesis and disrupting tumor cell metabolism (Figure 1). Furthermore, the review outlines future perspectives and current limitations of PDT, highlighting the challenges that remain in optimizing these strategies for clinical use. These insights not only provide a comprehensive overview of potential interventions, but also serve as a guide to effectively translating PDT into clinical oncology practice.

## 2 Photocatalytic treatment mechanisms

**FIGURE 2:**
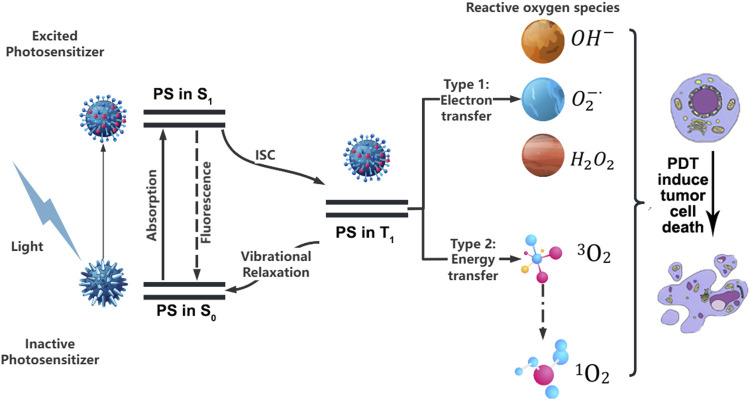
Schematic representation of photodynamic processes in tumor treatment. PS, Photosensitizer; S_0_, Ground state; S_1_, Excited singlet state; T_1_, Excited triplet state; ISC, Intersystem crossing.

PDT is a therapeutic method in which a photosensitizer (PS), stimulated by an external light source, converts oxygen inside the tumor cells into toxic ROS, which induces cell death by oxidizing intracellular biomolecules (Wang K. et al., 2022). Upon activation by light, the PS undergoes an energy transition from the ground state to a short-lived singlet state, which then undergoes intersystem crossing to a more stable triplet state (Jiang et al., 2023). From there, the PS can either release energy as heat or fluorescence, or interact with endogenous substances to generate free radicals, such as hydrogen peroxide and superoxide anion. The formation of ROS occurs through two mechanisms (Aebisher et al., 2024). Type I Mechanism, the PS participates in electron transfer processes, generating radicals. These radicals can react with oxygen to produce ROS such as superoxide anion (O_2_^−^), hydroxyl radical (OH**·**), and hydrogen peroxide (H_2_O_2_). Type II Mechanism, the PS transfers energy from its triplet state to molecular oxygen (^3^O_2_), resulting in the formation of highly reactive singlet oxygen (^1^O_2_), which is capable of inducing cell damage and death (Figure 2). Type I and type II reactions can be carried out simultaneously, and the ratio between them mainly depends on the photochemical and photophysical properties of PS and the concentrations of substrates and cellular oxygen (Yuan et al., 2021). Meanwhile, the two reactions can trigger different modes of cell death, in addition to the “conventional” cell death pathways such as apoptosis, necrosis and autophagy, other “unconventional” cell death modes such as ferroptosis and pyroptosis were also triggered. These findings provide new insights into the PDT-induced death signaling pathway (Mishchenko et al., 2022).

## 3 Alleviating tumor hypoxia and enhancing PDT efficacy

Despite the promising potential of PDT in tumor treatment, several drawbacks have emerged during its clinical application, garnering increasing attention in current research. The hypoxic tumor microenvironment, the existence of metabolic reprogramming defense mechanism, and abnormal tumor angiogenesis greatly limit the efficacy of PDT, and make the clinical application of PDT face great challenges. Firstly, most PDT processes are oxygen-dependent, which often leads to reduced efficacy when treating hypoxic tumors (Srivastava et al., 2023). Secondly, tumor hypoxia primarily arises from structural abnormalities and functional dysregulation of tumor blood vessels, which lead to insufficient oxygen supply, thereby limiting the overall efficacy of photodynamic therapy (Guo et al., 2023). Thirdly, tumor metabolic abnormalities, such as aberrant glycolysis, abnormal mitochondrial metabolism, significantly influence the efficacy of PDT (Lin et al., 2024).

In recent years, various strategies have been reported to improve tumor hypoxia with the aim of enhancing the efficacy of PDT. One of the strategies lies in the nano-delivery systems that carry oxygen to ameliorate the hypoxic microenvironment in tumors (Luo et al., 2018). Another significant consideration is the supply of oxygen, strategies focuses on *in situ* oxygen generation within tumors to alleviate oxygen deficiency (Gao et al., 2020). In addition to oxygenation strategies, some studies have applied oxygen-independent PDT mechanisms to improve treatment outcomes (Yi et al., 2020). Recent advancements in oxygen-independent PDT leverage nanomaterials that generate cytotoxic free radicals under hypoxia. For example, AIE-active phenanthroimidazole derivatives (AQPO and AQPI) with minimized singlet–triplet splitting, which shift ROS production from oxygen-dependent singlet oxygen (Type-II) to hypoxia-tolerant free radicals (Type-I), demonstrating enhanced therapeutic efficacy *in vivo* (Xiao Y.-F. et al., 2022). This approach not only bypasses hypoxia limitations but also disrupts redox homeostasis, amplifying metabolic stress in tumors. Manganese oxide nanosystems catalytically convert H_2_O_2_ to O_2_, addressing hypoxia while enhancing PS activation (Liu et al., 2019). Furthermore, Mn^2+^-coordinated PSs exhibit photocatalytic O_2_ regeneration, sustaining ROS production in deep tumor regions, these systems synergize with anti-angiogenic therapies by normalizing vessel permeability and reducing VEGF expression (Zuo et al., 2024). However, studies aimed at improving the tumor hypoxic microenvironment by intervening in tumor angiogenesis and metabolism to enhance PDT have not received adequate attention.

Extracellular matrix (ECM), a structure that not only supports blood vessel formation and stability, but also plays an important role in tumor growth and metastasis (Yao et al., 2020). The ECM is a critical regulator of tumor hypoxia and therapeutic resistance. Excessive ECM deposition increases tumor stiffness and interstitial fluid pressure, impairing both oxygen diffusion and photosensitizer penetration into tumors (Hu et al., 2020). Emerging strategies now prioritize ECM remodeling to alleviate hypoxia and amplify PDT efficacy. For instance, Li et al. designed ultrasound-responsive nanoparticles (UNPS) that generate ROS under hyperbaric oxygen to degrade collagen, thereby reducing ECM density. This dual-action approach enhanced oxygen perfusion and nanoparticle penetration, achieving superior PDT effects even at low laser doses (Li et al., 2018). Similarly, Cheng et al. developed a CAF-reprogramming nanomaterial (FPC@S) that degrades ECM proteins via localized ROS while releasing SIS3 to suppress CAF-driven ECM overproduction. This dual modulation normalized tumor vasculature and oxygenation, creating a favorable microenvironment for PDT (Qiu et al., 2024). Deformable nanomaterials like T-PFRT further address spatial barriers: MMP2-responsive size-shrinking enables ECM penetration, while TGF-β inhibition (via LY364947) prevents pathological ECM deposition. Combined with oxygen-loaded hemoglobin (OxyHb) and photosensitizers, this platform synergistically alleviates hypoxia and enhances ROS generation (Liang et al., 2021). Pancreatic ductal adenocarcinoma (PDAC) presents a unique challenge for PDT due to its dense desmoplastic stroma and hypovascular microenvironment, which severely limits nanomedicine penetration and oxygen availability. Recent studies have addressed this by developing stroma-targeting nanoplatforms. For instance, iron oxide-based nanoparticles conjugated with collagenase-I were shown to degrade extracellular matrix components in PDAC models, enhancing PDT efficacy by improving both photosensitizer delivery and oxygen perfusion (Shah et al., 2022). Similarly, in glioblastoma multiforme (GBM), where the blood-brain barrier (BBB) restricts drug accumulation, BBB-penetrating nanoparticles loaded with chlorin e6 and catalase demonstrated dual functionality: they alleviated hypoxia via H_2_O_2_ decomposition while achieving deep-tissue PDT activation under MRI guidance (Wu C. et al., 2024).

## 4 Emerging photodynamic nanotechnological approaches

Angiogenesis plays a pivotal role in tumorigenesis, serving as a prerequisite for tumor initiation and a foundation for its growth, invasion, and metastasis. Metabolic reprogramming refers to the process by which tumor cells alter their metabolic pathways to meet the demands of rapid proliferation and survival. Building on the identified challenges in PDT, this part explores emerging photodynamic nanotechnological approaches that demonstrate enhanced PDT efficacy, with key methodologies and comparative advantages summarized in Table 1.

**TABLE 1:** Intervention of tumor vasculature and metabolism enhanced photodynamic therapy.

Strategy type	Nanocomposites	Mechanism	Advantages	Limitations	References
Vascular normalization	LCL/ZnO	LCL/ZnO regulated the homeostasis of tumor vascular endothelial cells by activating TRPV4-eNOS signalling.	Selectively targeting tumor cells and multifunctional therapy	Effectiveness relies on tumor-specific H_2_O_2_ concentrations, which may vary across cancer types or stages.	Zhang et al. (2022a)
Vascular normalization	DES	Dexamethasone was used to normalize vascular function within the TME to reduce local hypoxia.	Enhanced tumor penetration and improved PDT efficacy	No data on patient-derived models and clinical trials	Zhu et al. (2020)
Vascular normalization	Erb-AAPS	Thalidomide to remodel the tumor vasculature and increase the tumor accumulation of the micelles.	Combined strategies significantly improve antitumor effects by synergizing nanoparticle accumulation and active targeting	Efficacy relies on tumor-specific TME features, which may vary across patients or cancer types.	Yan et al. (2021)
Vascular normalization	ECCaNPs	Erlotinib normalizes tumor blood vessels by inhibiting the EGFR/ERK/AKT axis to enhance intra-tumoral oxygenation.	pH-Responsive Release; Multifunctional Therapy; Biocompatibility and Targeting	Double-emulsion process may hinder scalability and reproducibility. Efficacy may vary in tumors with low EGFR expression or resistance mutations.	Liu et al. (2022)
Vascular normalization	PA7R@siPD-L1	A7R dissociated from the micelle complex targets VEGFR-2 and NRP-1 on tumor endothelial cells to normalize blood vessels.	Restructures chaotic tumor vasculature, alleviates hypoxia, and enhances immune-cell infiltration.	Multifaceted design (PDT + siRNA + peptide) complicates manufacturing and clinical translation.	Yi et al. (2022)
Vascular normalization	SPMI/3	PFKFB3 kinase inhibitor-based nanoplatforms normalized tumor vessels.	Tumor-Targeted Delivery; Combines PDT and PTT for potent early-stage tumor inhibition	Degradation pathways and byproducts of polydopamine (PDA) remain undefined, raising safety concerns.	Yu et al. (2024)
Intervention in Aerobic Glycolysis	C&S/Fe@S-S-OSCLMs	Sal-B-Fe alleviates tumor hypoxia by inhibiting aerobic glycolysis.	Focuses on cancer-specific glucose metabolism, improving treatment precision and sparing healthy tissues.	Efficacy relies on tumor GSH levels, which may vary across cancer types or stages.	Su et al. (2023)
Intervention in Aerobic Glycolysis	Polymer@Gef-YAP-siRNA NPs	Hypoxia was significantly alleviated by YAP-siRNA and GefHIF-1α expression and inhibition of glycolysis.	Combines targeted therapy (Gefitinib), gene therapy (YAP-siRNA), and photodynamic therapy (PDT) to overcome EGFR-TKI resistance in cancer.	Potential off-target effects and transient gene silencing require further optimization.	Huang et al. (2022)
Intervention in Aerobic Glycolysis	TCe6/CHC NPs	CHC regulates lactate metabolism to spare more intracellular oxygen for PDT.	TPGS modification improves Ce6’s solubility, stability, and cellular internalization. CHC alleviating tumor hypoxia.	Multi-step chemical coupling and co-assembly may hinder scalability.	Qin et al. (2021)
Intervention in Mitochondrial OXPHOS	ATO-IPS@NPs	The released ATO targets mitochondrial complex III to inhibit cellular respiration	Using low-oxygen-consuming Type-I photosensitizer (IPS), preserving intratumoral oxygen for enhanced PDT efficacy.	Tumor-specific OXPHOS activity variations may reduce ATO’s effectiveness in some cancers.	Zhang et al. (2022b)
Intervention in Mitochondrial OXPHOS	HAS-Ce6/NTZ/FCS NPs	The conventional anti-parasitic drug nitensamide (NTZ) regulates tumor cell metabolism to effectively improve tumor hypoxia.	Fluorinated chitosan (FCS) reversibly modulates bladder epithelium tight junctions, enabling efficient tumor penetration for intravesical PDT.	Self-assembled NPs may face aggregation or inconsistent drug release during storage or *in vivo*.	Wang et al. (2021)
Intervention in Mitochondrial OXPHOS	3BP@PLGA-IR780	3BP can effectively inhibit the oxygen consumption of tumor cells by inhibiting mitochondrial respiratory chain.	IR780 enables precise subcellular localization in mitochondria, enhancing ROS generation at the site of energy metabolism.	3BP’s efficacy depends on tumor glycolytic activity, which may vary across cancer types.	Wen et al. (2021)
Intervention in Glutamine Metabolism	BCH NPs	GLS inhibitor BPTES blocked the glutamine metabolic pathways by inhibiting GLS activity.	Enhancing redox imbalance for tumor-selective killing.	Glutamine-independent cancer cell subpopulations may evade therapy.	Yu et al. (2023)
Intervention in Glutamine Metabolism	C9SN	C968-mediated GSH deprivation through inhibiting glutamine metabolism prevented PDT-generated ROS from being annihilated by GSH	Amplifying intracellular oxidative stress and remodeled the ITM by blocking glutamine metabolism.	more work will need to be done to explore the inhibitory effect of C9SN on other tumor cells, especially those with lower glutaminase activity	Mai et al. (2023)
Intervention in Glutamine Metabolism	CeV	CeV can reduce the uptake of glutamine through V9302-mediated alanine-serine-cysteine transporter of type-2 (ASCT2) inhibition	Leading to a reduced glutathione (GSH) production and an amplified oxidative stress.	Efficacy relies on tumor glutamine addiction; resistant clones may bypass ASCT2 inhibition.	Zhao et al. (2022a)
Intervention in Cholesterol Metabolism	CPSA NPs	The SIM released from NPs could inhibit the HMGCR from reducing the cholesterol level in tumor tissue specifically	Ce6-mediated PDT/PTT induces immunogenic cell death (ICD), releasing tumor antigens to activate adaptive immunity and immune memory.	Chronic SIM use could induce myopathy or hepatotoxicity.	Song et al. (2023)
Intervention in Cholesterol Metabolism	EALP	AVA reprograms the immunosuppressive microenvironment by blocking cholesterol metabolism.	Amplifying antitumor immunity alongside IR780-induced immunogenic cell death.	Long-term toxicity and metabolic fate of MnO_2_ nanoparticles remain unverified.	Jian et al. (2022)

## 5 Targeted vascular enhanced photodynamic therapy

Abnormal vascular structures within the tumor microenvironment often lead to inadequate accumulation of photosensitizers, thereby weakening the therapeutic efficacy of PDT (Yue et al., 2021). Tumors subjected to low-dose PDT not only fail to eliminate tumor cells but also activate signaling pathway molecules crucial for tumor survival, thereby facilitating tumor survival and recurrence (Wan et al., 2021). Therefore, how to solve the obstacles of photosensitizer delivery, improve the uptake and aggregation of photosensitizer in the tumor, reduce tumor recurrence, and thus improve the efficacy of photodynamic therapy is a scientific problem that needs to be solved urgently. Recent strategies have focused on combining PDT with anti-angiogenic agents, offering a potential therapeutic synergy. However, growing evidence suggests that anti-angiogenic drugs are unable to sustain long-term inhibition of tumor angiogenesis, and do not significantly improve long-term survival outcomes (Broekgaarden et al., 2015). Furthermore, excessive anti-angiogenic treatment can exacerbate tumor hypoxia, impairing PDT efficacy (Loges et al., 2009). In recent years, some approaches have emerged to improve photodynamic antitumor efficacy, including the use of vascular normalization strategies.

### 5.1 Vascular interruption strategy

PDT-induced vascular damage acts as a double-edged sword in cancer therapy. The rapid generation of reactive oxygen species (ROS) during PDT induces endothelial cell apoptosis, platelet activation, and thrombosis, leading to vascular occlusion within minutes to hours post-treatment (Tao et al., 2022). While this “vascular shutdown” effectively starves tumors, surviving hypoxic cells at the tumor periphery often upregulate pro-angiogenic factors (e.g., VEGF, HIF-1α) and adopt aggressive metabolic phenotypes (e.g., enhanced glycolysis, lactate shuttling), driving recurrence and metastasis (Jung et al., 2021). To address this paradox, recent advances in photodynamic nanomedicine have focused on two synergistic strategies.

First, spatiotemporal control of vascular damage, nanoparticle-based PDT platforms (e.g., liposomes, polymeric micelles) enable precise spatial targeting of tumor vasculature while sparing normal tissues. For example, RGD peptide-modified nanoparticles selectively bind to αvβ3 integrins overexpressed on tumor endothelial cells, enhancing vascular disruption efficiency and reducing off-target effects (Javid et al., 2024). Additionally, light-triggered ROS generation from photosensitizers (e.g., chlorin e6) encapsulated in pH-responsive carriers allows controlled ROS release only within the acidic tumor microenvironment, minimizing premature vascular damage (Yang et al., 2018). Second, combinatorial vascular modulation, co-delivery of PDT agents with anti-angiogenic drugs (e.g., bevacizumab-conjugated nanoparticles) to prevent compensatory revascularization (Tangutoori et al., 2016). These strategies underscore the transformative potential of photodynamic nanomedicine in balancing acute vascular destruction with long-term metabolic regulation, ultimately mitigating relapse risks.

### 5.2 Vascular normalization strategy

**FIGURE 3:**
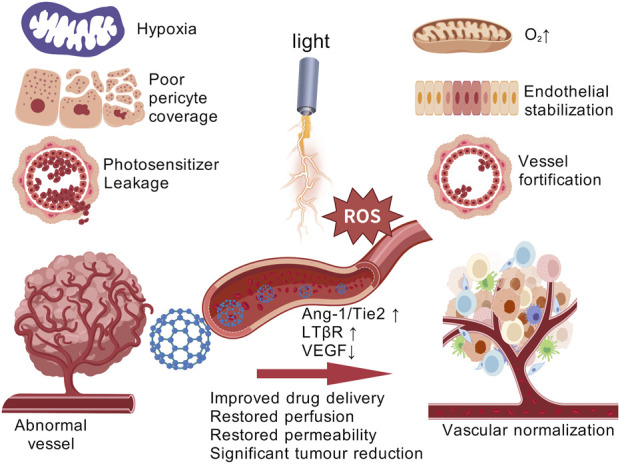
Vascular normalization strategies for enhancing PDT tumor therapy. [Created with BioGDP.com (Jiang et al., 2025)].

Tumors often exhibit rapid and aberrant angiogenesis, which results in malformed blood vessels that hinder the effective delivery of photosensitizers in PDT. Targeting these pathological blood vessels presents a rational therapeutic strategy (Yang et al., 2021). In 2005, Jain and colleagues introduced the concept of “vascular normalization,” suggesting that anti-angiogenic therapies, when administered within specific dosages and time windows, could restore balance in tumor vasculature, leading to more organized blood vessels, increased perfusion, and a temporary normalization of the tumor vasculature. This normalization alleviates tumor hypoxia, improves the delivery and accumulation of therapeutic agents, and enhances the overall therapeutic efficacy (Xiao M. et al., 2022). Vascular normalization has thus emerged as a promising approach to enhance PDT outcomes, ultimately improving PDT effectiveness (Figure 3).

A recent study reported a novel nanomaterial, SA-PEG-MPDA, which is loaded with indocyanine green and SPMI/3. After irradiation with 808 nm NIR light, this nanomaterial demonstrated significant antitumor efficacy. Moreover, SA-PEG-MPDA facilitated the normalization of tumor vasculature by increasing the coverage of laminin and pericytes, decreased the permeability of blood vessels, and improved the hypoxia of tissues, thus effectively enhancing the therapeutic effect of PDT (Xu et al., 2021). Previous studies have shown that the multi-targeted tyrosine kinase inhibitor, Lenvatinib, can inhibit vascular endothelial growth factor receptors (VEGFRs) and induce tumor vascular normalization (Liu et al., 2021). Tan et al. developed a Combo-NP, a nanomaterial loaded with lenvatinib. Upon near-infrared (NIR) light irradiation, the Combo-NP is cleaved by ROS, releasing lenvatinib. The released lenvatinib normalizes tumor blood vessels, reduces tumor hypoxia, and significantly enhances the effectiveness of photodynamic therapy (Zheng XQ. et al., 2023). Zhang et al. introduced a novel nanomaterial, LCL/ZnO, which demonstrates significant potential in tumor vascular normalization. LCL/ZnO promotes the normalization of abnormal tumor vasculature, reducing tumor hypoxia, and enhancing the overall therapeutic effect of PDT. Beyond merely normalizing tumor blood vessels, LCL/ZnO also extends the duration of the vascular normalization window, improving tumor vascular perfusion. The singlet oxygen (^1^O_2_) generated by LCL/ZnO activates signaling pathways in tumor-derived endothelial cells, including transient receptor potential vanilloid-4 (TRPV4) and endothelial nitric oxide synthase (eNOS), which contribute to vascular normalization (Zhang et al., 2022a).

## 6 Interfering with tumor cell metabolism to enhance photodynamic therapy

Metabolic reprogramming is often characterized by enhanced glycolysis, increased lipid synthesis, and alterations in amino acid metabolism, enabling tumor cells to thrive in nutrient-deprived and hypoxic environments. PDT has emerged as a promising treatment modality, where a photosensitizer generates ROS under specific light irradiation to selectively induce tumor cell death. However, the metabolic adaptability of tumor cells may profoundly influence the efficacy of PDT. Recent studies suggest that metabolic reprogramming can not only affect the uptake and activation of photosensitizers but may also contribute to tumor microenvironmental hypoxia, limiting ROS generation. Therefore, exploring the impact of tumor metabolic reprogramming on photodynamic therapy can help provide new ideas and strategies to improve the clinical outcomes of PDT. This section will focus on recent metabolic reprogramming strategies closely associated with PDT.

### 6.1 Modulating glycolysis to enhance photodynamic therapy

**FIGURE 4:**
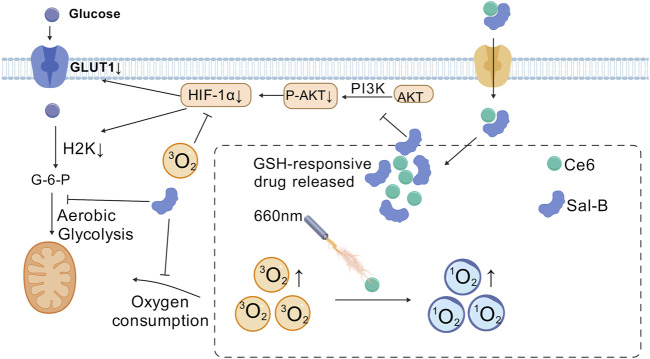
Salvianolic acid B (Sal-B) alleviate the tumor hypoxia through the inhibition of the aerobic glycolysis and improve the antitumor performance of PDT. [Created with BioGDP.com (Jiang et al., 2025)].

Aerobic glycolysis stands as one of the most extensively studied metabolic reprogramming phenomena within tumors. This process was first identified by the German scientist Warburg, who observed that tumor even in an environment rich in oxygen, preferentially engage in aerobic glycolysis to sustain the rapid proliferation (Ma and Zong, 2020). Glycolysis allows tumor cells to competitively consume large amounts of glucose to fuel their growth (Wu D. et al., 2024). As tumor cells enhance their aerobic glycolysis, they produce substantial amounts of metabolic byproducts. This results in an accumulation of lactate and exacerbates hypoxia within the tumor microenvironment. Furthermore, photodynamic therapy, which induces oxygen consumption and microvascular damage, further contributes to the local hypoxia in the tumor (Zhou Y. et al., 2022). This hypoxic condition drives additional glycolytic activity, creating a vicious cycle. Therefore, targeting tumor glycolysis to enhance the efficacy of PDT in treating hypoxic tumors presents a highly feasible and promising approach (Figure 4). For example, Zhao et al. developed a tumor-targeting nanomaterial C&S/Fe@S-S-OSCLMs to enhance PDT efficacy by specifically inhibiting glycolysis. The loaded Sal-B component selectively suppresses cancer-specific HK2 and GLUT1, reducing normal tissue damage while regulating tumor glucose metabolism. The Sal-B and Fe^3+^ complex releases under high glutathione (GSH) in tumors, simultaneously inhibiting aerobic glycolysis and decreasing oxygen consumption. This dual action alleviates tumor hypoxia and significantly improves PDT therapeutic outcomes (Su et al., 2023). Lactate, an inevitable product of glycolysis, is both an important metabolic energy source and a key gluconeogenic precursor and signaling molecule. Lactic acid acts as both an energy donor and signaling molecule, promoting tumor metastasis. Its extracellular accumulation creates an acidic, hypoxic tumor microenvironment, compromising therapeutic efficacy. The presence of lactate in the tumor microenvironment is closely related to biological behaviors such as tumor cell growth, metastasis, neovascularization, and immune escape (Bogdanov et al., 2022). Therefore, targeted inhibition of lactate metabolism may be an effective strategy to enhance photodynamic antitumor therapy (Tian et al., 2022). The nanomaterial Dc&Ce6@MBNP designed by Zhou et al. carries the photosensitizer Ce6 and the lactate dehydrogenase inhibitor diclofenac (Dc). This nanomaterial releases Dc during PDT for tumor treatment, inhibiting the activity and expression of LDHA, reducing lactate production, and further weakening tumor cell glycolysis and angiogenesis, thus enhancing the therapeutic effect of PDT (Zhou Y. et al., 2022). Zhao et al. introduced a novel self-delivery ternary bioregulators (named TerBio). The construction of TerBio is based on the self-assembly of a photosensitizer (Ce6), a TGF-β antagonist SB505124 (SB), and the anti-tumor drug lonidamine (Lon). The release of SB and Lon effectively reprograms the tumor microenvironment (TME), inhibits the TGF-β signaling pathway and lactate (LA) efflux, thereby reversing the immunosuppressive microenvironment and enhancing the photodynamic anti-tumor effect (Zhao et al., 2022b).

### 6.2 Intervention with mitochondrial OXPHOS to enhance photodynamic therapy

**FIGURE 5:**
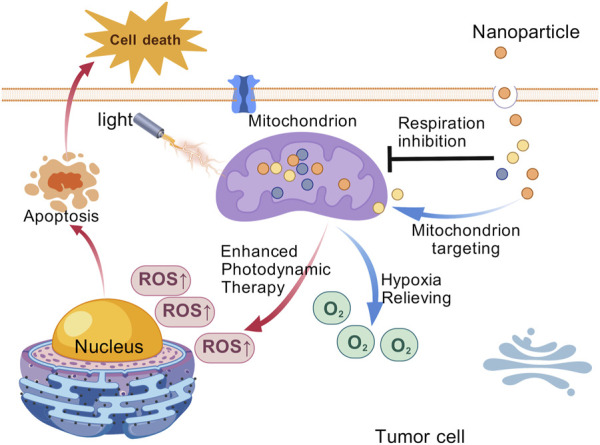
Schematic diagram of the mitochondrial targeting nanoparticles for efficient photodynamic therapy against the solid tumor. [Created with BioGDP.com (Jiang et al., 2025)].

Although tumor cells predominantly rely on glycolysis for energy production, mitochondrial oxidative phosphorylation (OXPHOS) also plays a crucial role. Numerous studies have shown that mitochondria can adapt to the metabolic demands of tumor cells, a phenomenon referred to as “mitochondrial plasticity” (Ward and Thompson, 2012). The mitochondrial OXPHOS, remains indispensable for tumor cell growth and proliferation. While most tumor cells rely on aerobic glycolysis, they also require OXPHOS to generate ATP. Some tumor cell can even switch flexibly from aerobic glycolysis to OXPHOS to meet energy demands (Smolkova et al., 2011). As the energy hub of the cell, mitochondria are the site of cellular respiration. Research has shown that mitochondrial-targeted PDT can effectively inhibit tumor ATP synthesis and reduce oxygen consumption, thereby reversing tumor hypoxia and accelerating tumor cell apoptosis (Zhao et al., 2020). Therefore, inhibition of mitochondrial OXPHOS could represent a promising strategy to enhance the efficacy of PDT (Figure 5). Wu et al. proposed a novel nanoparticle platform (TNPs/IA) designed to alleviate the hypoxic environment of tumors by targeting OXPHOS, which in turn enhances the efficacy of PDT. The nanomaterial enhances PDT through encapsulated atovaquone by inhibiting OXPHOS activity and reducing the oxygen consumption rate to alleviate tumor hypoxia, thereby improving ROS generation (Gao et al., 2024). Shen et al. developed MB@Bu@MnO2 nanoparticles to enhance PDT by targeting mitochondrial oxidative phosphorylation. The butformin (Bu) component inhibits tumor cell oxygen consumption, alleviating tumor hypoxia, while methylene blue (MB) generates cytotoxic ROS under laser irradiation to induce immunogenic cell death. This dual-action system synergistically improves therapeutic outcomes (Zhou Z. et al., 2022). Xu et al. proposed a multistage drug delivery nano-system for synergistic photodynamic therapy of tumors by sequential delivery of TPE-Py and RC. Mitochondria, the target organelles of TPE-Py, mediated the efficient aggregation of TPE-Py, and TPE-Py induced apoptosis and autophagy in tumor cells under light conditions. Meanwhile, Rubioncolin C, a natural naphthoquinone dimer, inhibited mitochondria-associated OXPHOS, which enhanced the PDT effect of TPE-Py by alleviating hypoxia (Wang J. et al., 2022).

### 6.3 Intervention in glutamine metabolism to enhance photodynamic therapy

**FIGURE 6:**
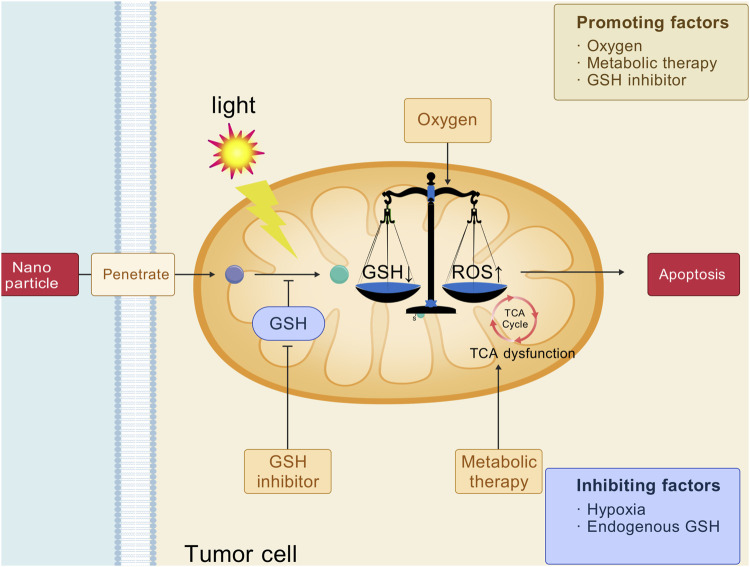
Strategies of intervention in glutamine metabolism for cancer photodynamic therapy. [Created with BioGDP.com (Jiang et al., 2025)].

Glutamine is a non-essential amino acid and a precursor of many biomolecules, providing energy for cell growth, maintaining cell redox homeostasis, and playing an important role in cell growth and proliferation. Tumor cells have a significant increase in the demand for glutamine to satisfy their energy needs for rapid growth (Scalise et al., 2017). Glutamine is converted by aminotransferase into α-ketoglutarate (α-KG), which enters the tricarboxylic acid cycle (TCA) to provide energy and synthesize precursors for tumor cells (Mates et al., 2020). In 2021, Rathmell et al. reported that tumor cells even preferred glutamine to glucose for energy metabolism (Reinfeld et al., 2021). In addition, glutamine is a precursor of GSH, which helps tumor cells counteract oxidative stress, thus promoting their survival and proliferation (Bansal and Simon, 2018). Blocking glutamine metabolism in tumor cells reduces reductive substances and may enhance oxidative antitumor effects of PDT (Byun et al., 2020). A variety of mechanisms associated with glutathione metabolism inhibition in tumors can enhanced the antitumor effect of PDT (Figure 6). In light of this, Zhao and colleagues developed a nanomaterial, CeV, to enhance the antitumor efficacy of PDT (Zhao et al., 2022c). CeV utilizes a self-assembly technique to combine chlorine e6 (Ce6) with V9302, creating a novel nanomedicine platform. V9302 inhibits the alanine-serine-cysteine transporter of type-2, which significantly reduces glutamine uptake in tumor cells. This mechanism decreases intracellular GSH levels, leading to increased oxidative stress, which further amplifies the antitumor effects of PDT. In another study, BVC nanoparticles enhanced photodynamic therapy by targeting glutamine metabolism, inhibiting glutamine transport and glutathione synthesis, and inducing tumor immunogenic cell death (Zhao et al., 2023).

### 6.4 Intervention in cholesterol metabolism to enhance photodynamic therapy

**FIGURE 7:**
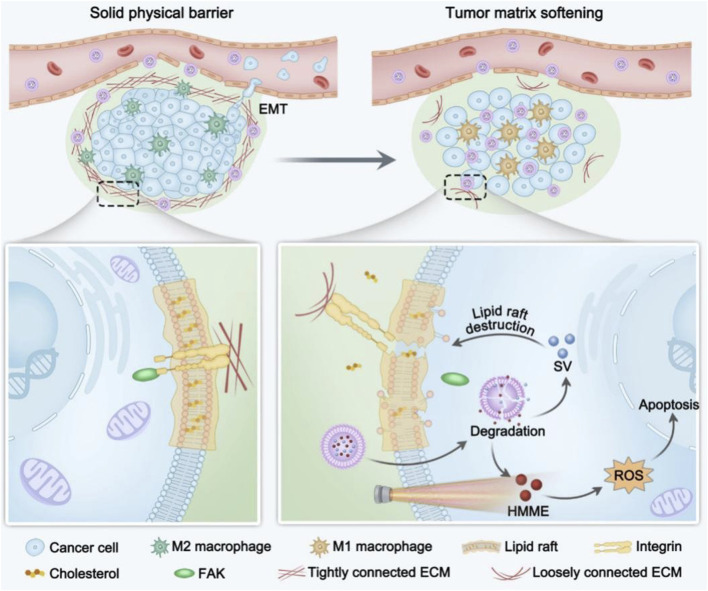
Scheme illustration of H/S@hNP-mediated cholesterol depletion strategy reduces tumor physical stiffness for enhanced photodynamic antitumor therapy (Peng et al., 2024).

Cholesterol is one of the essential molecules for eukaryotic cell survival and a key component of intracellular membrane structures. Cholesterol-derived metabolites also serve as important signaling molecules. Aberrant activation of the cholesterol biosynthetic pathway is a hallmark of many tumor, as it supports the rapid growth of tumor cells by providing the lipids required for cell membrane synthesis and facilitating the transmission of critical signaling pathways (Xue et al., 2020). Abnormal cholesterol metabolism in tumor cells is primarily characterized by increased cholesterol biosynthesis, enhanced exogenous cholesterol uptake, elevated cholesterol esterification and derivative production, as well as alterations in the tumor immune microenvironment (Chimento et al., 2018). In addition to directly influencing the biological behavior of tumor cells, cholesterol metabolism reprogramming also modulates the antitumor activity of immune cells within the tumor microenvironment. Cholesterol and its derivatives play a crucial role in the regulation of both innate and adaptive immunity, including processes such as monocyte expansion, neutrophil activation, NK cell activation, and T lymphocyte proliferation (Huang et al., 2020). Thus reprogramming cholesterol metabolism may enhance the therapeutic effect of PDT (Liu et al., 2023). H/S@hNP is a dual-drug-loaded nanoparticle that combines the photosensitizer hematoporphyrin monomethyl ether (HMME) and cholesterol synthesis inhibitor simvastatin (Figure 7). By depleting cholesterol, H/S@hNP blocks mechanical signaling between tumor cells and the ECM, thereby reducing the stiffness of the tumor stroma, facilitating drug penetration, and enhancing the efficacy of PDT (Peng et al., 2024). Zhang et al. designed a biological modulator, HN@CaCL-R, which successfully reversed the immunosuppressive state of the tumor microenvironment by regulating cholesterol metabolism, thereby enhancing the efficacy of PDT. HN@CaCL-R catalyzes the conversion of cholesterol to hydrogen peroxide (H_2_O_2_) within the tumor microenvironment, which enhances the luminescence of chemiluminescent reagent, further improving the effectiveness of PDT (Yin et al., 2023).

## 7 Limitations and clinical challenges of PDT

The limited tissue penetration of activating light remains a fundamental challenge in PDT. Traditional PDT relies on ultraviolet-visible (UV-Vis) light (400–700 nm), which is strongly absorbed by hemoglobin and melanin, restricting penetration to superficial tissues (Fan et al., 2016). Although near-infrared (NIR) light (700–1,100 nm) offers deeper penetration, its lower energy reduces reactive oxygen species (ROS) generation efficiency (Ethirajan et al., 2011). Strategies like upconversion nanoparticles (UCNPs) and X-ray-activated scintillators have been explored to enable indirect excitation of photosensitizers (PSs) in deep tissues (Chen et al., 2014).

Tumor hypoxia severely limits PDT efficacy, as oxygen is essential for ROS generation via Type II photochemical reactions (Li M. L. et al., 2020). Hypoxia not only reduces ROS production but also upregulates hypoxia-inducible factor 1α (HIF-1α), promoting tumor survival and angiogenesis (Cheng et al., 2016). To address this, self-oxygenating nanosystems and Type I PSs have been developed (Cheng et al., 2015). Despite promising preclinical results, clinical translation remains hindered by inconsistent oxygenation effects and potential off-target metabolic disruptions.

Compared to anti-angiogenic agents (e.g., bevacizumab), PDT shows unique advantages but faces distinct challenges. Bevacizumab (anti-VEGF) normalizes tumor vasculature, enhancing drug delivery but exacerbating hypoxia (Willett et al., 2004). In contrast, PDT transiently disrupts vasculature while inducing immunogenic cell death (ICD), potentially synergizing with checkpoint inhibitors (Zheng X. et al., 2023). Clinical trials combining PDT with bevacizumab demonstrated improved outcomes in colorectal cancer but highlighted risks of systemic toxicity (Peng et al., 2018).

## 8 Discussion

Malignant tumors are a leading global cause of death. While surgery remains primary treatment, current therapies for inoperable patients (e.g., organ dysfunction or advanced cases) often cause severe toxicities, highlighting the need for safer, more effective therapies. PDT shows promise but remains underdeveloped, hindered by tumor hypoxia, metabolic reprogramming, and abnormal vasculature. We review strategies targeting tumor vasculature and metabolism to enhance PDT efficacy and overcome clinical limitations.

The rationale for using nanomedicine in PDT lies in its ability to overcome critical limitations of traditional PDT while amplifying therapeutic efficacy through multifunctional design. First, nanocarriers enhance the delivery and bioavailability of hydrophobic PS, thereby improving solubility and prolonging circulation time for targeted accumulation in tumors (Rodrigues et al., 2020). Second, nanomedicine enables precise modulation of the tumor microenvironment, alleviating hypoxia and boosting ROS production during PDT (Di et al., 2023). Third, nanoplatforms facilitate combinatorial therapies, enabling organelle targeting to disrupt cancer cell metabolism and amplify ROS-induced apoptosis (Wu et al., 2023). Collectively, these strategies highlight pivotal role nanomedicine in optimizing PDT.

The concept of the tumor vascular normalization window—a transient phase post-anti-angiogenic therapy during which aberrant tumor vasculature acquires structural and functional stability—has emerged as a promising strategy to potentiate PDT. While preclinical studies demonstrate that synchronizing PDT with this window enhances therapeutic outcomes (Li Q. et al., 2020), its clinical translation remains hampered by the window’s elusive and transient nature. Current approaches rely on low-dose anti-angiogenic regimens to induce normalization, yet precise spatiotemporal control over drug delivery remains a critical unmet challenge (Shen et al., 2022). To address this, next-generation photosensitive nanomedicines are being engineered with three key features: (1) precision drug release to maintain tumor-localized, low-dose anti-angiogenic activity, thereby stabilizing vascular normalization; (2) sustained-release kinetics to prolong the therapeutic window; and (3) dose-tunable modulation to convert the indiscriminate “scissors” effect of anti-angiogenics into a “glue” effect that enhances pericyte coverage and vessel maturation (Shen et al., 2023).

Notably, the efficacy of PDT nanomedicines is further constrained by the tumor ECM, a dense physicochemical barrier that impedes nanoparticle penetration. While enzymatic ECM degradation, pharmacological inhibition, and hyperthermia-mediated ECM remodeling are under investigation, current strategies lack mechanistic depth. Most studies focus narrowly on collagen and hyaluronic acid, leaving other ECM components (e.g., fibrinogen, fibronectin) underexplored. Critically, no consensus exists on dominant signaling pathways governing ECM-PDT interactions, underscoring the need for multi-omics-driven target discovery and novel combinatorial approaches to modulate the tumor ECM-immune-metabolic axis.

Metabolic reprogramming, a hallmark of malignancy, presents another strategic lever to amplify PDT. While targeting tumor-specific metabolic vulnerabilities (e.g., dysregulated enzymes, nutrient transporters) holds potential, three key limitations persist: (1) incomplete mapping of tumor-specific metabolic networks due to gaps in single-cell metabolomics; (2) metabolic plasticity enabling therapeutic escape; and (3) off-target effects from metabolic inhibitors that disrupt normal cell physiology. To overcome these, next-generation PDT platforms must integrate dual/multi-pathway metabolic targeting—for instance, simultaneously inhibiting glycolysis and glutamine metabolism—to minimize compensatory adaptation. Furthermore, emerging “smart” nanomedicines with tumor microenvironment-responsive drug release could achieve selective metabolic targeting while sparing normal tissues. However, the interplay between tumor metabolism and immune cell function adds complexity: overzealous metabolic suppression may inadvertently cripple anti-tumor immunity. Thus, future designs must balance metabolic intervention with immune preservation, potentially through spatiotemporally controlled nano-delivery or orthogonal targeting of immune-metabolic checkpoints.

Despite these advances, clinical translation faces three major hurdles. First, the biocompatibility and long-term toxicity of multifunctional nanomaterials require rigorous evaluation using physiologically relevant models (e.g., orthotopic or patient-derived xenografts) rather than conventional subcutaneous tumors. Non-degradable nanoparticles (e.g., silica-based carriers) may accumulate in the liver/spleen, potentially causing chronic inflammation (Wei et al., 2024). Cationic lipid-based formulations can trigger complement-related hypersensitivity, as observed in failed Phase I trials of cationic porphyrin liposomes (Obaid et al., 2019). Second, the lack of standardized protocols for monitoring vascular normalization windows in humans necessitates innovative imaging biomarkers or liquid biopsy-based tools. Third, deeper mechanistic studies are needed to unravel how nanomaterials interact with tumor stroma, metabolism, and immune cells at single-cell resolution.

## 9 Conclusion and perspectives

The integration of photodynamic therapy (PDT) with nanotechnology has unlocked unprecedented opportunities for precision oncology. To inspire future developments, we outline key directions to expand the horizons of photodynamic nanomedicine in targeting tumor angiogenesis and metabolism. Develop stimuli-responsive nanocarriers that sequentially release anti-angiogenic drugs and activate photosensitizers under tumor-specific conditions (e.g., hypoxia, pH). Design “metabolic traps” that simultaneously achieve selective metabolic targeting while sparing normal tissues and enhance ROS generation through Fenton-like reactions. Integrate real-time oxygen sensors (e.g., phosphorescent porphyrins) into nanoplatforms to dynamically monitor and modulate TME oxygenation during treatment. The next-generation of photodynamic nanomedicine must embrace complexity—targeting angiogenesis and metabolism. By converging advances in materials science and systems biology, we can design multifunctional, patient-specific therapies that transcend traditional monotherapy limitations. Prioritizing translational studies and clinical-grade manufacturing will accelerate the transition from bench to bedside.

In conclusion, the convergence of vascular normalization, ECM remodeling, and metabolic reprogramming within PDT nanoplatforms represents a paradigm shift in oncology. By prioritizing tumor-selective multi-targeting, microenvironment-responsive drug release, and systems-level mechanistic validation, this strategy could transcend current limitations, ushering in a new era of precision photodynamic therapy.
